# Photoenhanced Radical Formation in Aqueous Mixtures of Levoglucosan and Benzoquinone: Implications to Photochemical Aging of Biomass-Burning Organic Aerosols

**DOI:** 10.1021/acs.jpca.3c01794

**Published:** 2023-06-07

**Authors:** Lena Gerritz, Meredith Schervish, Pascale S. J. Lakey, Tim Oeij, Jinlai Wei, Sergey A. Nizkorodov, Manabu Shiraiwa

**Affiliations:** Department of Chemistry, University of California, Irvine, California 92697-2025, United States

## Abstract

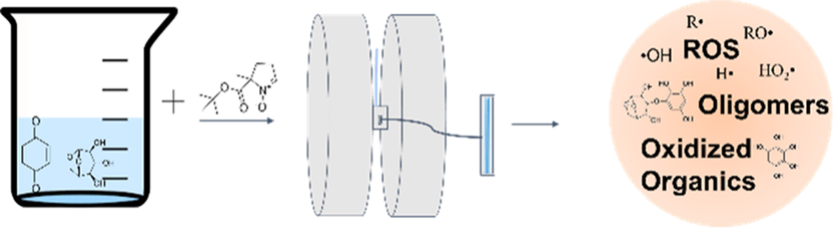

The photochemical aging of biomass-burning organic aerosols (BBOAs) by exposure to sunlight changes the chemical composition over its atmospheric lifetime, affecting the toxicological and climate-relevant properties of BBOA particles. This study used electron paramagnetic resonance (EPR) spectroscopy with a spin-trapping agent, 5-*tert*-butoxycarbonyl-5-methyl-1-pyrroline-*N*-oxide (BMPO), high-resolution mass spectrometry, and kinetic modeling to study the photosensitized formation of reactive oxygen species (ROS) and free radicals in mixtures of benzoquinone and levoglucosan, known BBOA tracer molecules. EPR analysis of irradiated benzoquinone solutions showed dominant formation of hydroxyl radicals (^•^OH), which are known products of reaction of triplet-state benzoquinone with water, also yielding semiquinone radicals. In addition, hydrogen radicals (H^•^) were also observed, which were not detected in previous studies. They were most likely generated by photochemical decomposition of semiquinone radicals. The irradiation of mixtures of benzoquinone and levoglucosan led to substantial formation of carbon- and oxygen-centered organic radicals, which became prominent in mixtures with a higher fraction of levoglucosan. High-resolution mass spectrometry permitted direct observation of BMPO-radical adducts and demonstrated the formation of ^•^OH, semiquinone radicals, and organic radicals derived from oxidation of benzoquinone and levoglucosan. Mass spectrometry also detected superoxide radical adducts (BMPO–OOH) that did not appear in the EPR spectra. Kinetic modeling of the processes in the irradiated mixtures successfully reproduced the time evolution of the observed formation of the BMPO adducts of ^•^OH and H^•^ observed with EPR. The model was then applied to describe photochemical processes that would occur in mixtures of benzoquinone and levoglucosan in the absence of BMPO, predicting the generation of HO_2_^•^ due to the reaction of H^•^ with dissolved oxygen. These results imply that photoirradiation of aerosols containing photosensitizers induces ROS formation and secondary radical chemistry to drive photochemical aging of BBOA in the atmosphere.

## Introduction

Biomass burning is one of the largest sources of organic aerosols and black carbon in the local and global atmosphere.^1^ Biomass-burning organic aerosols (BBOAs) also contain “brown carbon” that absorbs near-UV and visible radiation, contributing to positive radiative forcing.^1^ Brown carbon particles are hard to describe in air quality and climate models because they undergo photochemical transformations via functionalization, oligomerization, and fragmentation,^2^ resulting in dynamic evolution of their optical properties.^3−5^ Aging of BBOA by UV irradiation or hydroxyl radicals can result in an initial increase in absorption,^6−8^ which can be attributed to functionalization through hydroxylation, nitration, and oxidation as well as oligomerization, forming highly absorbing humic-like substances.^9−11^ After continued irradiation, many chromophores undergo fragmentation, resulting in a decrease in absorptivity, a phenomenon called “photobleaching”.^11−14^ Despite the recent surge to investigate how the chemical and optical properties of particles change with irradiation, mechanistic studies explaining the chemical changes during photochemical aging are scarce.^3^ One especially underexplored area is the role of reactive oxygen species (ROS) in the aqueous-phase chemistry of fresh and aged BBOA.^15^

ROS are defined to include hydroxyl radical (^•^OH), singlet oxygen (^1^O_2_), superoxide (O_2_^–•^), hydroperoxyl radical (HO_2_^•^), hydrogen peroxide (H_2_O_2_), ozone (O_3_), as well as carbon-centered and oxygen-centered organic radicals (R^•^/RO^•^).^15^ In the atmosphere, ROS are generated through photochemistry, gas-phase, and multiphase chemical processes, as well as fossil fuel combustion and biomass burning.^15−18^ ROS is an important aqueous-phase oxidant in aerosol chemistry, enhancing the formation and chemical transformation of secondary organic aerosols (SOA).^19−23^ Aqueous-phase generation of ROS also has important implications on toxicological properties as inhalation and respiratory deposition of BBOA in epithelial lining fluid and excess generation of ROS may induce oxidative stress, biological aging, cell death, and other health complications.^24^

Recent field measurements have found that chemical aging of BBOA increases its oxidative potential and leads to ROS generation.^25−27^ One known source of ROS in BBOA is quinones, a byproduct of incomplete combustion and fuel pyrolysis. Quinones are strong chromophores and can act as photosensitizers,^28^ which initiate photochemical processes within particles.^29^ These processes are driven by triplet-excited states of photosensitizers, and in the presence of other organic molecules, they can produce low-volatility organic compounds that contribute to SOA formation.^30−34^ The aqueous photochemistry of the simplest quinone, 1,4-benzoquinone (BQ), is shown to produce both hydroxyl and organic radicals,^35,36^ although ^•^OH formation has not been rigorously quantified. BBOA particles are a complex mixture of organic compounds, and to this point, no studies have explored the effect of other compounds on the photochemistry of BQ.

This work uses simplified BBOA surrogate mixtures of 1,4-benzoquinone (BQ) and levoglucosan (LVG) to explore the effect of constituents of BBOA that are stable with respect to direct photolysis on the photochemistry of much more reactive quinones in the aqueous phase. LVG is a known tracer compound derived from the combustion of cellulose and is considered as a ubiquitous and innocuous component of BBOA.^37^ Previous studies suggested that heterogeneous or aqueous oxidation of LVG by ^•^OH and NO_3_^•^ can shorten its chemical lifetime substantially, suggesting that LVG may not be the inert tracer molecule as traditionally considered.^38−43^ Since the irradiation of BQ produces aqueous ^•^OH, we expect it to accelerate degradation of LVG through condensed-phase chemistry. This study investigates the aqueous-phase photochemical aging of mixtures of BQ and LVG using electron paramagnetic resonance (EPR) with a spin-trap technique for in situ radical detection, high-resolution mass spectrometry (HRMS) for radical adduct identification, and kinetic modeling for investigating ROS formation kinetics and mechanisms.

## Experimental Methods

Mixtures were prepared by dissolving BQ (Sigma-Aldrich, >98%) and LVG (Sigma-Aldrich, 99%) in Milli-Q water. The final BQ and LVG concentrations were 0.5 to 4 and 2.5–50 mM, respectively, to obtain ratios of BQ/LVG of 1:0, 1:1, 1:10, or 1:100. The resulting solutions had a pH of ∼5 throughout the course of irradiation (not controlled). The solvents were exposed to room air and contained dissolved oxygen.

### EPR Experiments

Radical species were quantified using a continuous-wave EPR (CW-EPR) spectrometer (Bruker, Germany) with a spin-trapping technique. Prior to analysis, the spin-trap agent 5-*tert*-butoxycarbonyl-5-methyl-1-pyrroline-*N*-oxide (BMPO) (Enzo Life Sciences, ≥99%) was added to each aqueous mixture with a final BMPO concentration of ∼10 mM. The high concentration was intended to favor trapping of radicals by BMPO, but even at this concentration, some ^•^OH may be lost before being trapped by BMPO due to the high reactivity of ^•^OH. A 50 μL aliquot of the final mixture was then added to a 50 μL capillary tube and sealed prior to insertion into the resonator of the EPR spectrometer. Spectra were recorded with a center field of 3515.0 G, a sweep width of 100.0 G, a receiver gain of 30 dB, a modulation amplitude of 1.0 G, an attenuation of 12 dB, a microwave power of 12.6 mW, a microwave frequency of 9.84 GHz, a modulation frequency of 100 kHz, a conversion time of 5.12 ms, and a time constant of 0.01 ms.

For in situ UV irradiation experiments, the solution was irradiated using a UV irradiation system (ER203UV, Bruker), which delivered radiation from a 100 W Hg lamp through a 300 nm long-pass filter, a safety shutter connected to the EPR resonator, and a liquid light guide. Prior to the experiment, the lamp was warmed up for at least 10 min to stabilize its intensity. The actual spectral flux (shown in Figure S1) was characterized using a spectroradiometer (PS-200, Apogee Instruments) positioned at the same distance (2.5 cm) from the light guide as that to the sample in the EPR resonator. At the start of the EPR analysis, a dark EPR scan of the sample was performed with the safety shutter closed. The safety shutter was opened at the start of the second scan to start irradiation while continuing to record EPR spectra using the 2D time-delay method. For most experiments, the samples were continuously irradiated for 55 min, and spectra were averaged over 100 s every 2 min to obtain a high enough signal to noise. As the ^•^OH radical appeared promptly, experiments were also carried out with a finer time resolution, wherein the samples were irradiated for about 6 min, recording spectra approximately every 15 s, in order to capture the BMPO–OH formation before it began decaying.

The spectra were analyzed using the SpinFit and SpinCount programs built into the Bruker Xenon software. For more complex spectra, the EasySpin open-source software (https://www.easyspin.org/) was used to supplement the fitting parameters generated by SpinFit. The parameters used to generate the simulated spectra are reported in Table S1, and the radicals were quantified using the SpinCount function for the simulated spectra.^44^ To compare samples with different BQ concentrations, the samples were normalized to the total number of photons absorbed by BQ during irradiation to determine the radical yield. This number was calculated by using the experimentally obtained lamp spectrum (Figure S1), the wavelength-dependent absorption cross sections of BQ,^45^ and assuming that the overall concentration of BQ molecules, including its hydroquinone and hydroxylated derivatives, remained constant over the course of irradiation. We note that the normalization to BQ molecules is expected to be valid only at early irradiation times as BQ was promptly depleted in the experiment (see the Kinetic Modeling Results).

### Mass Spectrometry Analysis

Prior to HRMS analysis, 1 mL of a 1:10 BQ/LVG surrogate mixture (with or without added BMPO) was irradiated for 5 min. The radiation from the light guide (Figure S1) was directed from above into open 1.5 mL microcentrifuge tubes filled with 1 mL of the solution. The mixture was then diluted to an LVG concentration of 400 μg/mL in 50% v/v of acetonitrile in water. The same analysis was also performed on the nonirradiated mixture, irradiated BQ solution, and irradiated LVG solution as controls.

The chemical composition of the irradiated surrogate mixtures was analyzed using HRMS following the approach of Klodt et al., 2022.^46^ The mixtures were separated via ultrahigh-performance liquid chromatography (UPLC) using an HSS T3 Waters Acquity Premier 150 × 2.1 mm column with 1.8 μm particles. The UPLC solvent gradient started with 95% solvent A (water acidified to pH 3 using 0.1% formic acid) and 5% solvent B (acetonitrile acidified to pH 3 using 0.1% formic acid) from 0 to 3 min. From 3 to 14 min, the solvent linearly increased to 95% solvent B and 5% solvent A, where it remained constant from 14 to 16 min before a linear decrease back to 95% solvent A and 5% solvent B from 16 to 22 min. Following UPLC, the separated mixture was detected using a Thermo Q Exactive Plus mass spectrometer (Thermo Scientific), with a resolving power of 1.4 × 10^5^ at *m*/*z* 400. The parameters for the heated electrospray ionization (ESI) were as follows: capillary temperature, 325 °C; capillary voltage, +4.0 kV; sheath gas flow rate, 35; auxiliary gas flow rate, 10; sweep gas flow rate, 8; S-lens RF level 30; auxiliary gas heater temp, 300 °C. Negative ion mode mass spectra were also recorded but turned out to be less informative.

The HRMS data were analyzed using Freestyle 1.3 (Thermo Scientific) to integrate the total ion chromatogram (TIC) from 1 to 16 min to generate a raw time-integrated mass spectrum. The peak positions and relative abundances were extracted using Decon2LS software. The peaks from blank, unirradiated, and irradiated (both with and without BMPO) mixtures of 1:10 BQ/LVG surrogate mixtures were clustered using an *m*/*z* tolerance of 0.0005. Peaks containing ^13^C, as well as peaks present in the blank at the same or greater abundances as the samples were discarded. The remaining peaks were assigned to formulas C_*c*_H_*h*_O_*x*_N_0–2_Na_0–1_^+^ using a mass accuracy of *m*/*z* 0.0005. Peaks with abnormal Kendrick mass defects (generally, above 0 for the CH_2_ defect and below 0 for the O defect) were also removed because they were assumed to come from impurities.

### Kinetic Modeling of BQ Photochemistry

**Figure 1:**
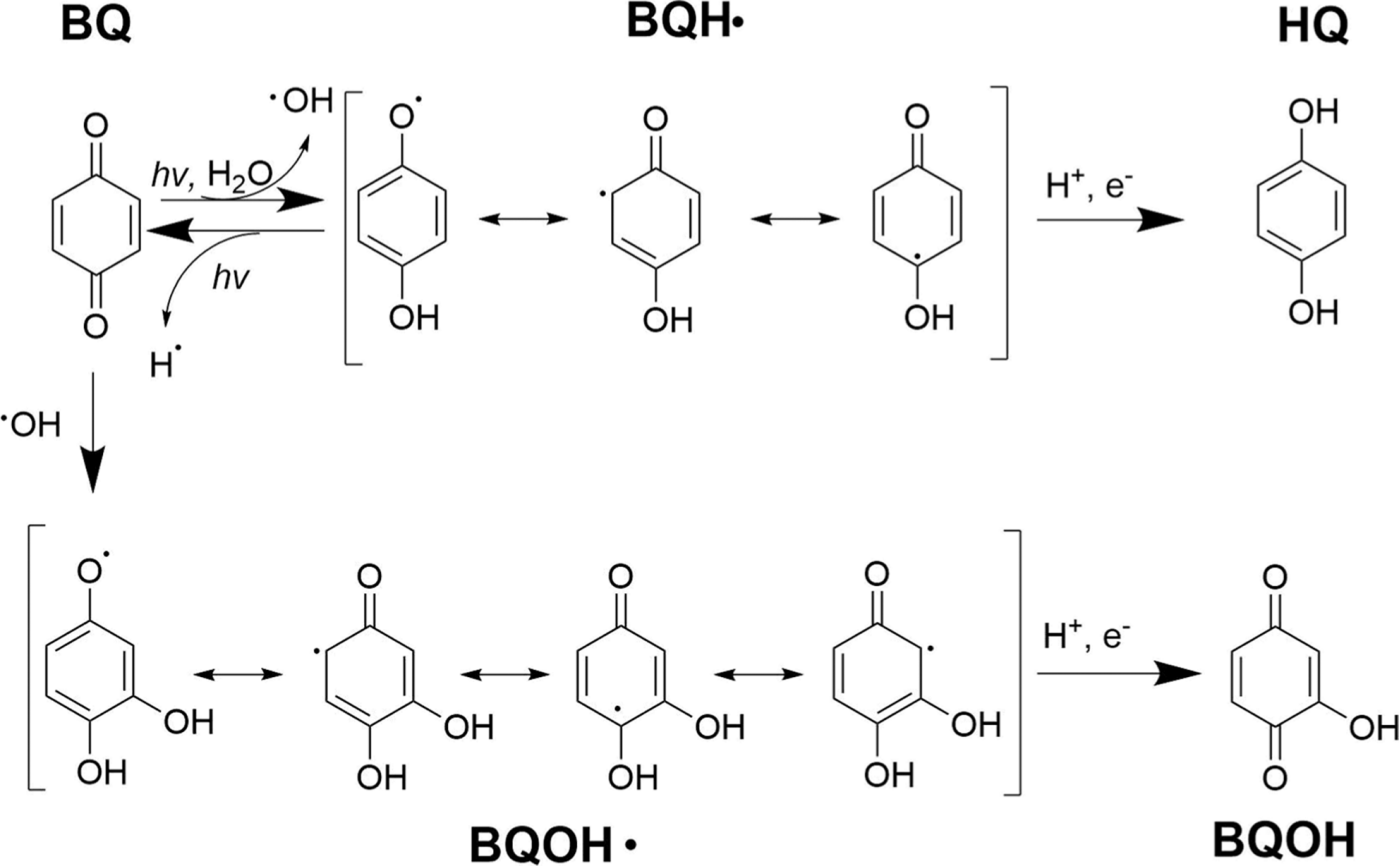
Structures of BQ and its photolysis products discussed in this study.

We modeled the kinetics of the BMPO–OH and BMPO–H adduct formation during irradiation of BQ, LVG, and BMPO in water using rate equations derived from the reaction mechanism presented in Table 1. The BQ photochemistry products discussed in this section are shown in Figure 1. The photochemistry begins with the absorption of a photon by BQ to excite the molecule into a singlet excited state (^1^BQ*) that rapidly undergoes intersystem crossing to create the reactive triplet-state molecule, ^3^BQ* (R1–R3). This ^3^BQ* is known to extract a hydrogen atom from water to produce ^•^OH and semiquinone radicals (BQH^•^) (R4).^35,36^ The resulting ^•^OH can then react with a ground-state BQ molecule to form a hydroxylated semiquinone radical (BQOH^•^) or get trapped by BMPO and be detected by EPR as BMPO–OH (R5 and R23). BQOH^•^ can undergo a self-reaction that terminates the radical propagation, generating BQOH and BQ (R8). It can also react with BMPO and undergo an ^•^OH abstraction to form BMPO–OH and BQ (R24).^35,36^ In order to explain the formation of BMPO–H, the mechanism detailed by Ononye et al. (1986)^35,36^ was amended based on a computational study proposing that BQH^•^ can photochemically decompose into BQ and H^•^ (R7).^47^ This H^•^ can react with dissolved oxygen (O_2_) producing HO_2_^•^ (R11), with ^•^OH forming water (R12), or with BMPO producing the BMPO–H adduct detected by EPR (R26).^48,49^ Reactions between organic radicals and dissolved oxygen are too slow compared to the rates of reactions of these radicals with BMPO. Other secondary reactions involving organic radicals are also excluded because they are not differentiable using EPR. This provides no EPR data for each individual organic radical that could be used in the model and therefore limits our ability to accurately parameterize their role in this system. This limitation should not impact the overall viability of the model as the concentrations of organic radicals are too low to affect the processes being explicitly treated in the model. The concentration of dissolved oxygen was not depleted by photochemistry based on the control measurements described in the Supporting Information. Additionally, each of the BMPO adducts can undergo dissociation reactions on a timescale of minutes to hours, but the exact mechanisms are poorly understood, so the decomposition reactions are lumped as one pathway for each adduct (R25, R27, and R30). The radicals in the solution also interact with each other through ROS coupling reactions (R13–R22). We also include LVG reactions with ^•^OH (R9) and ^3^BQ* (R10) to explain the effect of LVG concentration on the ROS formation of the mixture.

**Table 1:** Reaction Mechanism and Rate Constants Used in the Kinetic Model

Rxn no.	reaction	*k* (best fit)	units	*k* (literature)
Aqueous Irradiation Chemistry				
R1	BQ + *hv* → ^1^BQ*	1.71 × 10^–^^2^	s^–1^	9.7 × 10^–^^3^ a
R2	^1^BQ* → ^3^BQ*	3.60 × 10^12^	s^–1^	>5 × 10^10^ ^52^
R3	^3^BQ* → BQ	5.10 × 10^6^	s^–1^	1.9 × 10^6^ ^52^
R4	^3^BQ* + H_2_O→ ^•^OH + BQH^•^	1.56 × 10^9^	s^–1^	>1 × 10^7^ ^35,36^
R5	^•^OH + BQ → BQOH^•^	2.70 × 10^10^	M^–1^ s^–1^	1.1 ± 0.8 × 10^10^ ^31^
R6	BQOH^•^ + BQ → BQOH + BQH^•^	9.82 × 10^9^	M^–1^ s^–1^	2 × 10^7^ ^52^
R7	BQH^•^ + *hv* → BQ + H^•^	5.59 × 10^–^^6^	s^–1^	calculated^51^
R8	2BQOH^•^ → BQOH + BQ + H_2_O	9.03 × 10^7^	M^–1^ s^–1^	∼10^8^ ^35,36^
LVG Reactions				
R9	^•^OH + LVG → products	4.34 × 10^9^	M^–1^ s^–1^	6.0 to 38.0 × 10^9^ ^38^
R10	^3^BQ* + LVG → products	7.53 × 10^8^	M^–1^ s^–1^	n/a
Hydrogen Radical Chemistry				
R11	H^•^ + O_2_ → HO_2_^•^	fixed^b^	M^–1^ s^–1^	1.26 × 10^10^ ^53^
R12	H^•^ + ^•^OH → H_2_O	fixed	M^–1^ s^–1^	1.2 × 10^10^ ^48^
ROS Coupling Reactions				
R13	HO_2_^•^ → H^+^ + O_2_^•–^	fixed	s^–1^	2.3 × 10^5^ ^54,55^
R14	H^+^ + O_2_^•–^ → HO_2_^•^	fixed	M^–1^ s^–1^	1.74 × 10^10^ ^54,55^
R15	^•^OH + O_2_^•–^ → O_2_ + OH^–^	fixed	M^–1^ s^–1^	7.8 × 10^9^ ^56^
R16	^•^OH + ^•^OH → H_2_O_2_	fixed	M^–1^ s^–1^	5.2 × 10^9^ ^57^
R17	^•^OH + HO_2_^•^ → H_2_O + O_2_	fixed	M^–1^ s^–1^	7.2 × 10^9^ ^57^
R18	H_2_O_2_ + ^•^OH → H_2_O + HO_2_^•^	fixed	M^–1^ s^–1^	3.3 × 10^7^ ^58^
R19	H_2_O_2_ + HO_2_^•^ → H_2_O + O_2_ + ^•^OH	fixed	M^–1^ s^–1^	3.0^59^
R20	H_2_O_2_ + *hv* → ^•^OH + ^•^OH	fixed	s^–1^	2.7 × 10^–4^ ^51^
R21	HO_2_^•^ + HO_2_^•^ → H_2_O_2_ + O_2_	fixed	M^–1^ s^–1^	8.4 × 10^5^ ^60^
R22	HO_2_^•^ + O_2_^•–^ → H_2_O_2_ + OH^–^ + O_2_	fixed	M^–1^ s^–1^	1.0 × 10^8^ ^60^
BMPO Trapping Chemistry				
R23	BMPO + ^•^OH → BMPO–OH	5.07 × 10^8^	M^–1^ s^–1^	6.0 × 10^7^ ^54^
R24	BMPO + BQOH → BMPO–OH + BQ	2.47 × 10^6^	M^–1^ s^–1^	6 × 10^6^ ^35,36^
R25	BMPO–OH → products	1.65 × 10^–^^3^	s^–1^	n/a
R26	BMPO + H^•^ → BMPO–H	2.00 × 10^8^	M^–1^ s^–1^	n/a
R27	BMPO–H^•^→ products	7.44 × 10^–^^4^	s^–1^	n/a
R28	BMPO + HO_2_^•^ → BMPO–OOH	fixed	M^–1^ s^–1^	1.51 × 10^7^ ^54^
R29	BMPO + O_2_^•–^ + [H^+^]→ BMPO–OOH	fixed	M^–1^ s^–1^	2.41 × 10^7^ ^54^
R30	BMPO–OOH → products	2.8 × 10^–^^3^ (fixed)	s^–1^	2.2–9.5 × 10^–^^3^ (see Supporting Information)

a This value was calculated from the absorption cross sections and spectral flux density.

b These parameters were fixed at previously reported values.

The rate constants for reactions 1–10 and 23–27 were determined using the Monte Carlo genetic algorithm (MCGA) to fit the measured EPR signals for BMPO–OH and BMPO–H. The MCGA uses random sampling to produce sets of values for all the variable rate constants and uses the differential rate equations and reaction conditions to simulate the experimental data.^50^ Literature values were used to set the bounds for the model with a range of ±2 orders of magnitude, but the actual values were not fixed to allow the model to optimize rate constants based on the experimental data obtained. The fitness of each set of rate constants was evaluated using a least-square regression and the model outputted the rate constants for the best-fit set for each simulation. The uncertainty in rate coefficients was determined using the standard deviations of rate constants produced by 24 iterations of the MCGA with the same input bounds. The rate constants for R1, R7, and R20 were calculated based on the absorption cross sections of BQ and H_2_O_2_,^51^ along with the spectral flux of the light source (Figure S1). The rate constants calculated for R1 and R7 were used to set input bounds, but the rate constants reported in the table were found using the MCGA, while the rate constant for R20 was fixed at the calculated value. The rate constants for R11–R19, R21–R22, and R28–R29 were fixed based on literature values as EPR did not detect any BMPO–OOH, the trapped form of HO_2_^•^ and O_2_^–•^, in these measurements. A pH of 5 was assumed for all pH-dependent reactions.

## Results and Discussion

### Radical Composition

**Figure 2:**
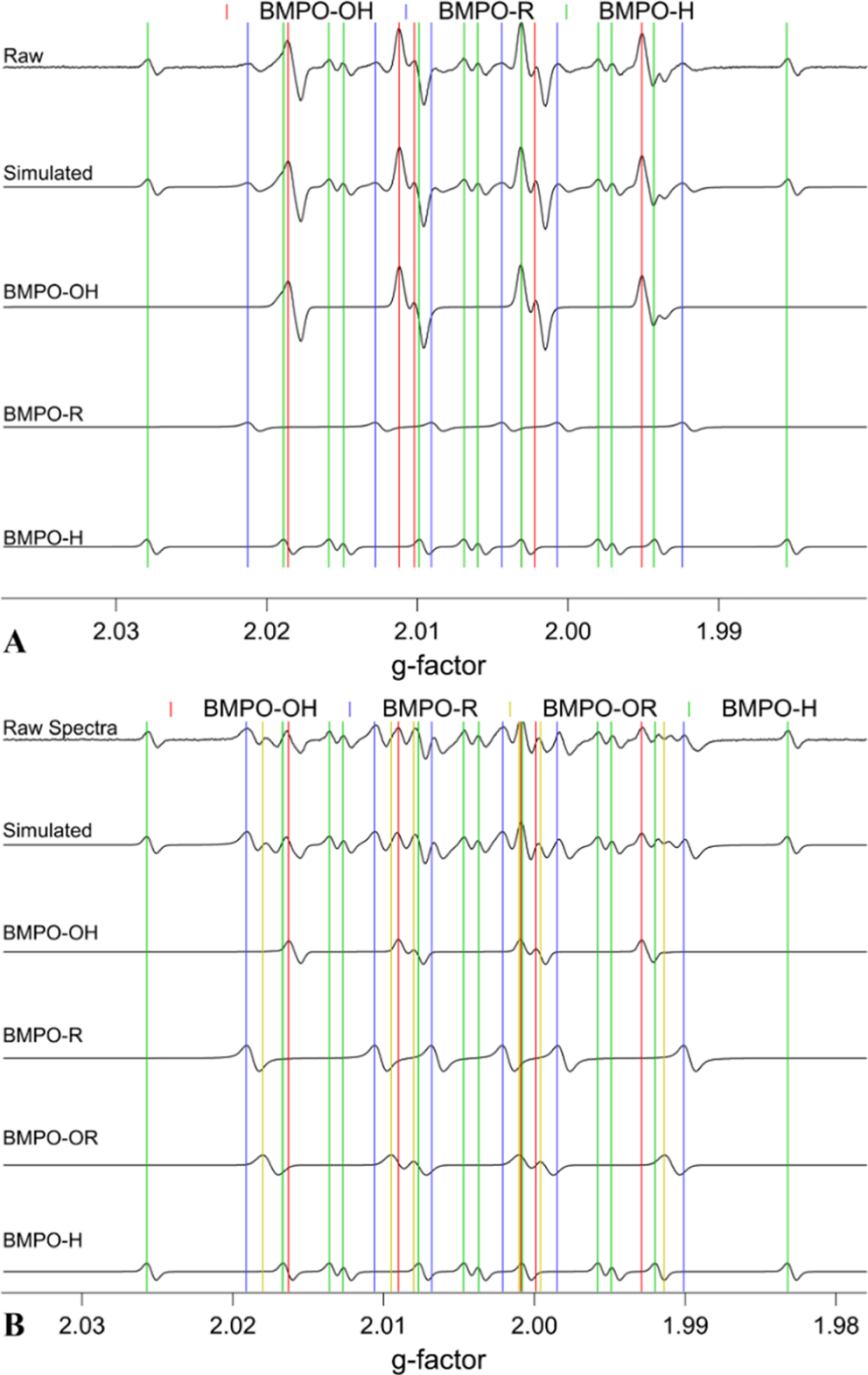
EPR spectra of (A) BQ solutions and (B) 1:10 mixture of BQ/LVG after 53 min of irradiation. The observed spectra were deconvoluted into BMPO-radical adducts: BMPO–OH (red), BMPO–R (blue), BMPO–OR (yellow), and BMPO–H (green).

The irradiation of surrogate BBOA mixtures generated substantial ROS detected by EPR. Figure 2 shows the EPR spectra of the BQ solution and the 1:10 BQ/LVG mixture after irradiating them for 53 min. The observed spectra were simulated and deconvoluted into four different BMPO-radical adducts including hydroxyl radicals (BMPO–OH), hydrogen radicals (BMPO–H), carbon-centered organic radicals (BMPO–R), and oxygen-centered organic radicals (BMPO–OR). The deconvoluted spectra for the 1:1 and 1:100 BQ/LVG mixtures are qualitatively similar to those of the 1:10 mixture (Figure S2). The irradiated BQ solutions mainly generated OH radicals with minor contributions from carbon-centered radicals and hydrogen radicals, while the formation of oxygen-centered organic radicals was also observed for BQ–LVG mixtures. This EPR analysis cannot resolve the chemical identity of the organic radicals in the BMPO–R and BMPO–OR adducts. The residual spectra after fitting showed a minimal difference between the raw and simulated spectra, and no adduct corresponding to BMPO–OOH was detected, implying that superoxide is not responsible for the observed prompt OH formation.

**Figure 3:**
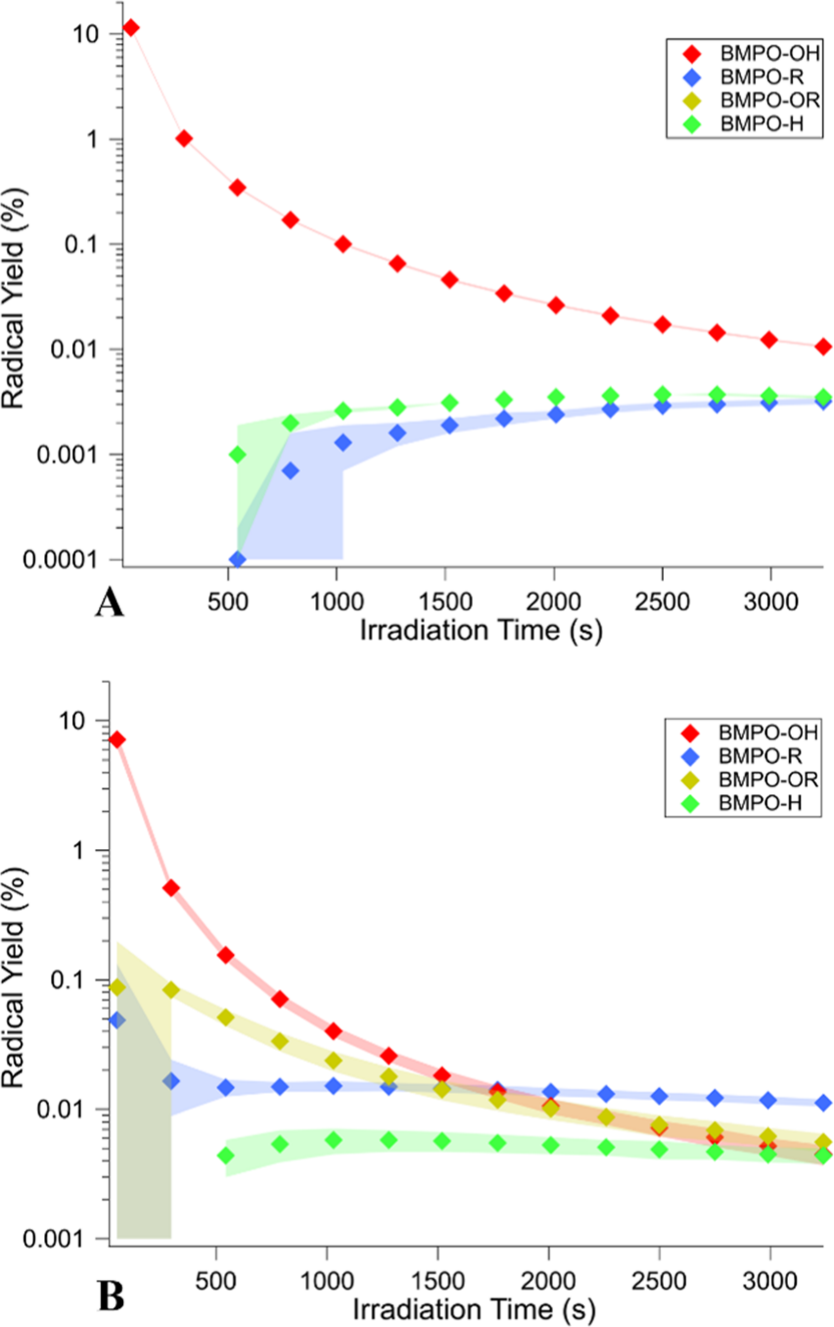
Radical yield (the number of produced radicals normalized to the number of photons absorbed by BQ) over irradiation time for (A) BQ solutions and the (B) 1:10 BQ/LVG mixture trapped using ∼10 mM BMPO. Shaded regions represent one standard deviation of the measurements. Irradiation begins at time *t* = 0.

Figure 3 shows the effective radical yields in irradiated BQ solutions and the 1:10 BQ/LVG mixture as a function of irradiation time. The radical yields are defined as the number of radicals trapped as a function of time (shown in Figure S3) divided by the total number of photons absorbed by BQ since the irradiation started. The irradiation produces an immediate spike in BMPO–OH for both samples, reaching their maxima within 30 s of irradiation. The concentrations of BMPO–R, BMPO–H, and (when applicable) BMPO–OR increase following the burst of ^•^OH, indicating that they are formed by secondary chemistry rather than direct photolysis.

Both the normalized (Figure 3) and non-normalized (Figure S3) data point to the prompt formation of ^•^OH followed by secondary chemistry, leading to the growth of the observed radicals. This is consistent with the proposed ^•^OH generation through direct reactions with ^3^BQ* as there is no time delay between irradiation and radical formation as would be expected of secondary reactions.^35,36,47,52^ We build on these studies by studying the temporal formation of the radical as well as its behavior in the presence of LVG, both of which have not been previously explored.

Previous studies have assumed that the organic radicals formed during the irradiation of aqueous BQ are oxygen-centered semiquinone radicals.^35,36,47^ The EPR results in Figure 2A are not consistent with this assertion because only BMPO–R adducts were detected in the absence of LVG. This suggests that either the carbon-centered resonance structure is more prevalent than the oxygen-centered resonance structure for BQH^•^ shown in Figure 1 or the carbon-centered structure is more readily trapped by BMPO. At higher concentrations of LVG, we did observe the formation of BMPO–OR radicals, suggesting that oxygen-centered radicals can form by secondary processes. However, due to the rapid interconversion pathways between ^•^OR/^•^R for resonance-stabilized molecules, EPR data alone are insufficient for determining whether the observed oxygen-centered radicals are derived from BQ or LVG.

The most novel result from this EPR analysis is the formation of BMPO–H. The mechanism was proposed in a computational study, but this is the first experimental evidence of H^•^ formation from the irradiation of BQ.^47^ H^•^ should be reasonably reactive, so in the absence of BMPO, it would react at the diffusion limit with dissolved oxygen and other radical species including ^•^OH, R^•^, and ^•^OR, creating a variety of secondary products, including HO_2_^•^ and organic peroxy radicals.^48^ These reactions are explored in more detail below using the kinetic model.

### Effect of BQ/LVG Ratios on Aqueous Chemistry

**Figure 4:**
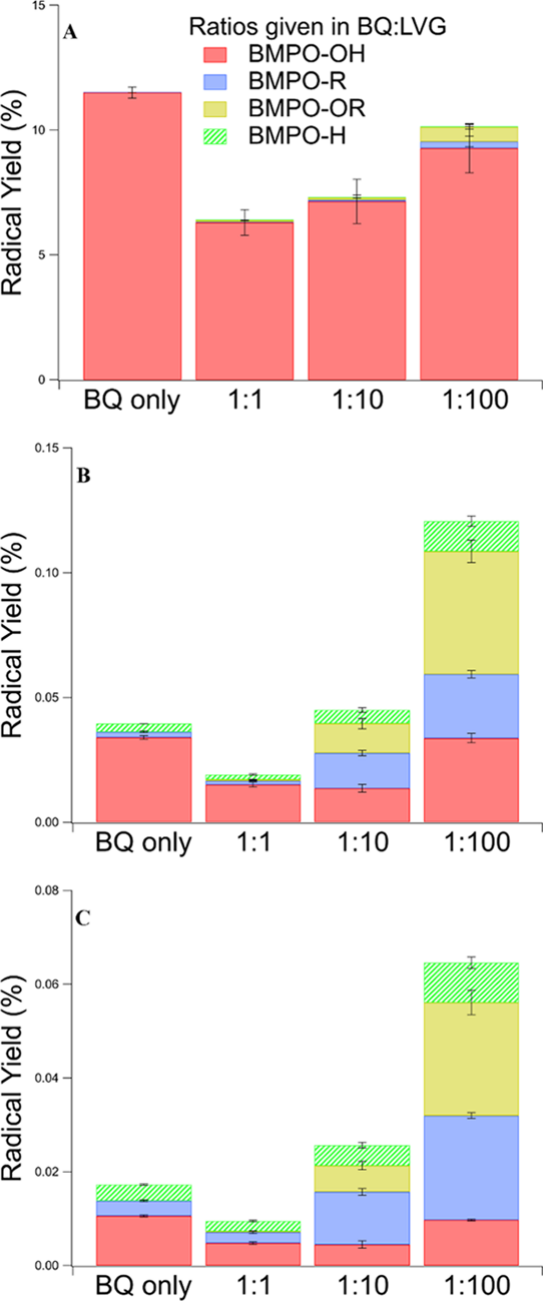
Radical yields (normalized to photons absorbed by BQ) for each solution: BQ only, BQ/LVG mixture with the ratio of 1:1, 1:10, and 1:100 after (A) 1 (beginning), (B) 29 (halfway), and (C) 53 min (end) of irradiation. Error bars represent one standard deviation of the measurements.

The role of LVG on the ROS formation of surrogate mixtures was investigated using four different molar ratios of BQ/LVG (1:0, 1:1, 1:10, and 1:100) by observing the EPR signal over time. Control experiments with irradiating solutions containing only LVG were also conducted, but no EPR signal was observed. Figure 4 shows the radical yields after 1, 29, and 53 min of irradiation (as mentioned above, the yields are only meaningful at early irradiation times due to swift decay of BQ, but they afford a convenient way to qualitatively compare relative abundances of different radicals at different BQ/LVG ratios). The most noticeable change in the presence of LVG is the detection of oxygen-centered organic radicals (BMPO–OR), in addition to carbon-centered organic radicals observed in the solution with only BQ. Additionally, the total organic radical concentrations (BMPO–R + BMPO–OR) increased with an increase in LVG, further confirming its indirect involvement in ROS formation. In contrast, the BMPO–H yield does not depend on the amount of added LVG, within the range of uncertainty. This suggests that the H^•^ formation can be fully attributed to the direct photodissociation of semiquinone radicals.^47^

**Figure 5:**
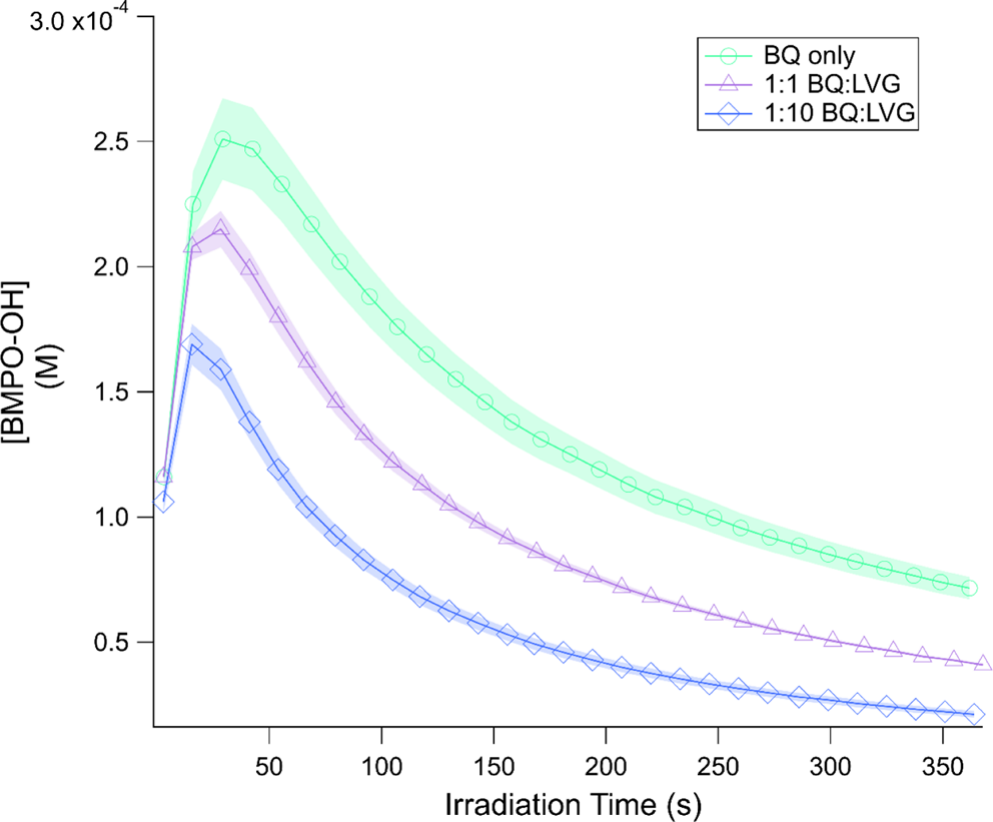
BMPO–OH formation over time for solutions of BQ only and 1:1 and 1:10 BQ/LVG mixtures with initial [BQ] = 2.5 mM and ∼10 mM BMPO. Shaded regions represent one standard deviation of the measurements. Irradiation begins at time *t* = 0.

In order to gain more quantitative information about the impact of LVG on BMPO–OH formation, additional irradiation experiments with a finer time resolution were performed on solutions of BQ only, 1:1 BQ/LVG, and 1:10 BQ/LVG with the same absolute concentration (2.5 mM) of [BQ], as shown in Figure 5. These experiments show that as the concentration of LVG increases, the BMPO–OH concentration decreases. This is most likely due to the increased rate of reaction between ^•^OH and LVG, competing with the reaction between BMPO and ^•^OH to decrease the amount of BMPO-trapped ^•^OH. This is also consistent with the faster formation of BMPO–R/OR radicals at higher concentrations of LVG. This confirms that in complex mixtures, ^•^OH participates in secondary chemistry that leads to the aqueous-phase aging of the solutions.

### Mass Spectrometry Results: BMPO Ionization Pathways

To our knowledge, there is no previous literature describing the ESI mechanisms of BMPO-radical adducts. There are a few studies on ESI of a related spin-trapping compound 5,5-dimethyl-1-pyrroline *N*-oxide (DMPO), which may exhibit similar ionization pathways.^61,62^

We observe that BMPO adducts are readily ionized in the positive ion mode of ESI-MS, but the ionization mechanism is more complex than that for a typical closed-shell organic compound. Each BMPO adduct is detected at multiple masses, and several possible ionization pathways (Figure S4) can be inferred from our analysis of the observed mass spectra. The mass spectrum observed at the retention time corresponding to BMPO (Figure S5A) shows three major peaks. The peak at *m*/*z* 200.129 in Figure S5A corresponds to unfragmented BMPO ionized via protonation to form [(BMPO) + H]^+^, while the peak at *m*/*z* 399.249 corresponds to a protonated BMPO + BMPO ion cluster [BMPO + BMPO + H]^+^. These are expected peaks for the positive ion mode ESI, but they are not the strongest: the dominant peak, *m*/*z* 144.066, is a fragmentation peak that corresponds to the loss of the *tert*-butyl group from the protonated BMPO ([(BMPO)-C_4_H_8_]^+^). This fragmentation pathway, which is promoted by the elevated temperature of the capillary in the mass spectrometer ion source, also occurs for most BMPO-radical adducts, leading to the presence of two peaks, corresponding to the same parent complex. This is exemplified by BMPO–OH, which is detected as [(BMPO–OH)-C_4_H_8_]^+^ and [(BMPO–OH)]^+^ to produce peaks B and F, respectively. In some cases, both of them are not major peaks, in which case only one is highlighted on the spectrum. (Note that we use a long dash in the notation for the radicals (BMPO–X) and ± sign in square brackets to denote the gain or loss of atoms during the ionization process.)

The ionization of BMPO–radical adducts is even more complicated than that of BMPO itself as they do not undergo standard protonation. In addition to the fragmentation pathway mentioned above, there are also three other possible ionization mechanisms inferred from the mass spectral analysis. The three pathways observed are depicted in Figure S4B and correspond to *m*/*z* equal to the molecular weight (MW) of the BMPO adduct ([(BMPO–X)]^+^) (1a/2a), *m*/*z* equal to the MW of the BMPO adduct plus 2 ([(BMPO–X) + H + H]^+^) (1b/2b), and *m*/*z* equal to the MW of BMPO adduct plus 24 ([(BMPO–X) + H + Na]^+^) (1c/2c). The first pathway (1a/2a), which is the most prominent, results from the formation of a double bond between the nitrogen and carbon atoms while adding a proton to the oxygen to create a positive charge on the nitrogen, as previously described for DMPO, a similar spin trap molecule.^61^ The other two pathways require a multistep mechanism to form a reduced BMPO–X compound, similar to a mechanism previously proposed for DMPO.^62^ The first step is the attachment of a hydrogen atom to the free radical site in the BMPO–X adduct resulting in a nonradical species. This likely occurs prior to ionization as a radical quenching mechanism and must also contribute to the BMPO–X signal decay observed in time-resolved EPR spectra. This quenched species can then undergo standard protonation to form [(BMPO–X) + H + H]^+^ or form a sodium complex, [(BMPO–X) + H + Na]^+^, both of which would be regarded as standard pathways for positive-ion-mode ESI.

While the presence of three interconnected ionization pathways substantially increases the complexity of the mass spectra of spin-trap complexes, it still provides important insight into the chemical nature of the radicals being formed. For example, the directly ionized [(BMPO–H)]^+^ adduct cannot be distinguished from the protonated [(BMPO) + H]^+^ (peak D, *m*/*z* 200.129). However, peak E (*m*/*z* 202.14) corresponds to a BMPO molecule with three added hydrogen atoms. This can only be formed through a BMPO–H adduct that was quenched by a second hydrogen prior to MS analysis, resulting in a neutral adduct that then underwent normal H^+^ ionization to produce the [(BMPO–H) + H + H]^+^ peak, unequivocally proving the presence of hydrogen radicals in the irradiated mixture. The Na^+^ ionization pathway can provide similar information because the formula of the compound can be unambiguously obtained by removing the Na atom from the observed ion formula. This enables the identification of adduct formulas for peaks O and T by confirming that there is one additional hydrogen that is not a part of the adduct. This results in formulas corresponding to [(BMPO–BQ) + H + Na]^+^ (peak O) and triply hydroxylated [(BMPO–BQ(OH)_3_) + H + Na]^+^ (peak T), thus clearly distinguishing them from hydroquinone/hydroxylated hydroquinone [(BMPO–HQ)]^+^/[(BMPO–HQ(OH)_3_)]^+^ which have the same *m*/*z* as [(BMPO–BQ) + H + H]^+^/[(BMPO–BQ(OH)_3_) + H + H]^+^, respectively.

### Mass Spectrometry Results: Radical Adduct Identification

The major radical adducts detected using HRMS are summarized in Table 2 and labeled in Figure S5D. The most abundant peaks in the mass spectra correspond to the unadducted BMPO and its derivatives (peaks A, D, and V) as well as BMPO–OH radicals (peaks B and F). This is expected as BMPO is in a large excess in the solution and BMPO–OH is the most abundant adduct detected by EPR. Additionally, the mass spectrum revealed a relatively small BMPO–OOH adduct (peak C) not discernible by EPR because of its relatively low concentration. The HO_2_^•^ radical is expected to form from the reaction between H^•^ and dissolved oxygen and will be discussed further in the kinetic model section.

**Table 2:** Peak Identifications for the Mass Spectra

peak	*m*/*z*	relative abundance	chemical formula	adduct identification^a^
A	144.066	100	C_6_H_10_O_3_N^+^	[(BMPO)-C_4_H_8_+H]^+^ or [(BMPO–H)-C_4_H_8_]^+^
B	160.060	55.5	C_6_H_10_O_4_N^+^	[(BMPO–OH)-C_4_H_8_]^+^
C	176.056	4.60	C_6_H_10_O_5_N^+^	[(BMPO–OOH)-C_4_H_8_]^+^
D	200.129	15.1	C_10_H_18_O_3_N^+^	[(BMPO) + H]^+^ or [(BMPO–H)]^+^
E	202.144	0.06	C_10_H_20_O_3_N^+^	[(BMPO–H) + H + H]^+^
F	216.124	0.73	C_10_H_18_O_4_N^+^	[(BMPO–OH)]^+^
G	250.071	0.02	C_12_H_12_O_5_N^+^	[(BMPO–BQ)-C_4_H_8_]^+^
H	252.087	3.88	C_12_H_14_O_5_N^+^	[(BMPO–BQ)-C_4_H_8_ + H + H]^+^ or [(BMPO–HQ)-C_4_H_8_]^+^
I	266.066	0.30	C_12_H_12_O_6_N^+^	[(BMPO–BQOH)-C_4_H_8_]^+^
J	300.071	0.01	C_12_H_14_O_8_N^+^	[(BMPO–HQ(OH)_3_)-C_4_H_8_]^+^
K	304.103	0.19	C_12_H_18_O_8_N^+^	[(BMPO–LVG)-C_4_H_8_]^+^
L	308.149	3.94	C_16_H_22_O_5_N^+^	[(BMPO–BQ) + H + H]^+^ or [(BMPO–HQ)]^+^
M	310.165	0.03	C_16_H_24_O_5_N^+^	[(BMPO–HQ) + H + H]^+^
N	326.160	0.25	C_16_H_24_O_6_N^+^	[(BMPO–HQOH)]^+^
O	330.131	0.74	C_16_H_21_O_5_NNa^+^	[BMPO–BQ) + H + Na]^+^
P	342.155	0.04	C_16_H_24_O_7_N^+^	[(BMPO–HQ(OH)_2_) + H + H]^+^
Q	358.149	0.01	C_16_H_24_O_8_N^+^	[(BMPO–HQ(OH)_3_) + H + H]^+^
R	360.165	0.52	C_16_H_26_O_8_N^+^	[(BMPO–LVG)]^+^
S	378.176	0.03	C_16_H_28_O_9_N^+^	[(BMPO–LVG(O)) + H + H]^+^
T	382.147	0.17	C_16_H_25_O_8_NNa^+^	[(BMPO–BQ(OH)_3_) + H + Na]^+^
U	392.155	0.01	C_16_H_26_O_10_N^+^	[(BMPO–LVG(O_2_))]^+^
V	399.249	10.2	C_20_H_35_O_6_N_2_^+^	[(BMPO–BMPO) + H]^+^

a In this notation, the adducts between BMPO and free radicals are placed in the round brackets and have a long dash. The processes that happen to the adduct in the ionization sources are encoded using the standard mass spectrometric notation in the square brackets. For example, [(BMPO–X)]^+^ represents a directly ionized adduct, while [(BMPO–X) + H]^+^ represents the same adduct that was protonated.

The most valuable information from the mass spectrometry analysis is the speciation of the organic radicals at a level that is not possible with EPR (Table 2). We have observed BMPO adducts derived from both benzoquinone (peaks G, H, L, M, and O) and LVG (peaks K and R). In the presence of excess ^•^OH, BQ is known to undergo continuous hydroxylation, promoted by the addition of electron-donating OH substituents which lead to the highly hydroxylated BQ derivatives trapped by BMPO (peaks I, J, N, P, Q, and T).^9^ Additionally, the oxidation products of LVG (peaks S and U) confirm that both compounds participate in aqueous radical chemistry initiated by the BQ photolysis. This further proves that the presence of innocuous compounds, represented by LVG in surrogate mixtures, can contribute to the organic radical chemistry of solutions in the presence of photosensitizers.

**Figure 6:**
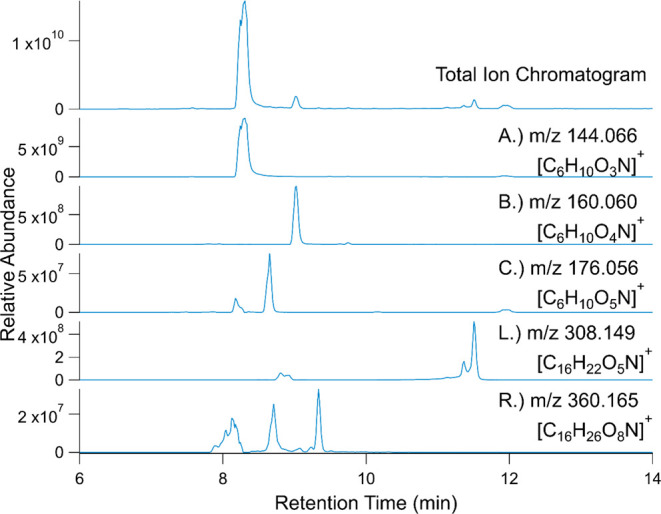
LCMS chromatograms for the irradiated 1:10 BQ/LVG mixtures with 10 mM BMPO including the TIC and selected ion chromatograms for select BMPO adducts featured in Table 2.

For additional information about the composition of the 1:10 BQ/LVG irradiated mixture with BMPO, the TIC and selected ion chromatograms with retention times between 6 and 14 min for select BMPO adducts are shown in Figure 6. The TIC contains three distinct peaks at 8.3, 9.0, and 11.5 min as well as a broad peak around 12 min.

The most dominant peak at 8 min corresponds to the elution of the BMPO, which is detected as a BMPO fragment (ion labeled A in Table 2 with *m*/*z* 144.066), unfragmented BMPO (D), and the BMPO dimer (V). The large height of the BMPO peak agrees with the experimental design that BMPO should be in excess of any radicals formed during irradiation. The peak at 9 min corresponds to BMPO–OH adduct, which ionizes primarily into BMPO–OH fragment (ion labeled B in Table 2 at *m*/*z* 160.060) with minor contributions from unfragmented BMPO–OH (F) and sodiated BMPO–OH. The observation of this peak agrees with the EPR measurements showing that BMPO–OH is a major component of the radical mixtures.

While EPR results detected no measurable contributions from BMPO–OOH (Figure 2), it is possible to observe it by HRMS due to its higher sensitivity. Figure 6 shows the selected ion chromatogram corresponding to the BMPO–OOH fragment (ion labeled C in Table 2 at *m*/*z* 176.056). Since there is only one possible structural isomer of BMPO–OOH, we can expect one peak in the chromatogram; however, there are three well-resolved peaks at 8.2, 8.7, and 12 min. All of these peaks are not visible in TIC, consistent with the negligible yield of BMPO–OOH based on EPR measurements. In the absence of standards, we presume that the largest of the three peaks eluting at 8.7 min corresponds to BMPO–OOH, while the two weaker peaks at 8.2 min and 12 min are larger compounds that produce ions with the same formula during ionization.

The TIC peak eluting around 11.5 min correlates with ion labeled L in Table 2 at *m*/*z* 308.149, which can be produced by ionization of either (BMPO–BQ) or (BMPO–HQ) adducts. The height of this peak in TIC is comparable to that from BMPO–OH, suggesting that it is a dominant organic radical formed in the mixture. The 11.5 min peak is split into two likely due to the presence of different structural isomers of the radical adduct corresponding to the location of the free radical site on BQ. There is also a small doublet eluting around 9 min, suggesting that more than two isomers are possible. The ability to resolve different isomers is a powerful feature of the HRMS-based method in comparison to the EPR observations that cannot be used to speciate different types of organic radicals beyond being carbon- or oxygen-centered.

Figure 6 also provides strong evidence for existence of multiple isomers of BMPO–LVG adducts. The selected ion chromatogram for the ion labeled R at *m*/*z* 360.165 has at least three separate peaks. The relative abundance of these peaks is not high enough to contribute to the TIC, in agreement with the much lower yield of LVG-derived radicals compared to ^•^OH based on the EPR data (Figure 4). The exact structure of these isomers cannot be determined in the absence of suitable standards but could be an interesting area of future research as different radical site locations in LVG likely exhibit different reactivities that could be important for secondary radical chemistry.

The mass spectrum corresponding to the 12 min peak contains a number of ions with the *m*/*z* above 400 corresponding to BMPO oligomers as well as unfragmented BMPO (peak D) and its fragment (peak A). The longer retention time compared to that for smaller BMPO adducts suggests that species eluting at 12 min are formed prior to entering the column and are not artifacts of ionization of BMPO itself. These compounds do not provide significant new information about the adducts formed and therefore will not be discussed further.

The irradiated mixture of BQ and LVG without BMPO was also analyzed by HRMS. The time-integrated mass spectrum (Figure S5C) was rather complex, suggesting that there are potentially hundreds of product peaks formed during irradiation when compared to the nonirradiated mixture (Figure S5B). Notably, there are many peaks with *m*/*z* values corresponding to dimers and trimers of BQ and/or LVG, as well as an increase in the average O/C ratio after irradiation, both of which are important factors in promoting aqueous-phase SOA formation.^9^ In addition to the higher-MW compounds formed after irradiation, there are also a variety of compounds with lower molecular weights that do not directly align with functionalized products of BQ and LVG, implying that BQ and/or LVG fragmentation reactions also take place during irradiation (although we cannot separate it from the effect of fragmentation in the ion source). These low-molecular-weight compounds would support previously proposed ring cleavage decomposition pathways for BQ,^63^ but future work needs to be conducted to investigate the role of LVG on the fragmentation mechanism.

### Kinetic Modeling Results

**Figure 7:**
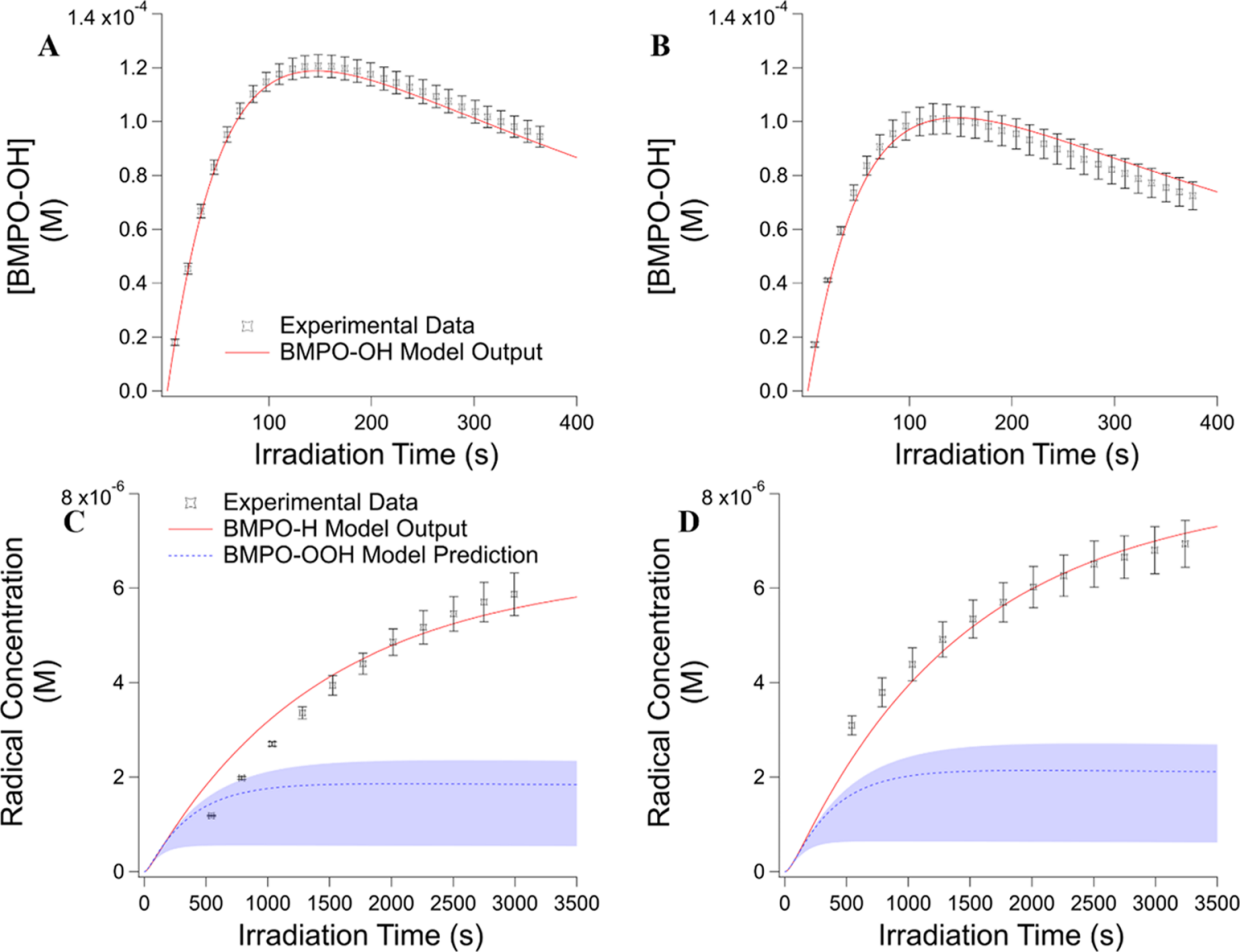
Observed (markers) and modeled (red lines) time evolution of BMPO–OH concentrations (A) in the BQ solution and (B) in the 1:10 BQ/LVG mixture over 400 s of irradiation as well as the time evolution of BMPO–H concentrations and the model-predicted BMPO–OOH concentrations (C) in the BQ solution and (D) in the 1:10 BQ/LVG mixture over 3500 s of irradiation. The shaded regions represent the model uncertainties from the rate constant of R30. Irradiation begins at time *t* = 0.

As shown in Figure 7, the kinetic model was able to simultaneously fit the formation of BMPO–OH and BMPO–H, in solutions with and without LVG, to the experimental EPR data using the reaction scheme in Table 1. The result of the kinetic model supports the proposed mechanism for BMPO–OH and BMPO–H formation and is extended to explore the implications of HO_2_^•^ formation in the system.

The mechanism of BMPO–OH formation from the irradiation of benzoquinone is well constrained based on previous experiments (R1–R6, R8, R23, and R24), so the primary goals of modeling these reactions were to obtain rate constants that were optimized to fit the BQ/BMPO system by accounting for the BMPO–H formation observed in this work and the effects of added LVG. Past studies provided approximate rate constants for many of the radical reactions, and the rate constants determined by this model agree within an order of magnitude with those previously reported, except for R6 (BQOH^•^ + BQ → BQOH + BQH^•^). The discrepancy for this rate constant (MCGA and literature values of 9.82 × 10^9^ and 2 × 10^7^ M^–1^ s^–1^, respectively) is most likely due to the interconversion between quinone (BQOH^•^, BQ^•^) and semiquinone (BQOH^•–^, BQ^•–^) states that naturally exist in the solution but are indifferentiable using EPR. The reaction of the conjugate forms, BQOH^•–^ + BQ → BQOH + BQ^•–^, was not treated as a separate step from R6 in the model mechanism because of the rapid interconversion between the two forms. This conjugate reaction has a rate constant of 2 × 10^9^ M^–1^ s^–1^, which agrees with the output of MCGA.^52^ This should not impact the overall model as the interconversion is fast enough so that the two reactions can be grouped under one step for the sake of BMPO–OH formation analysis. The reasonable agreement between the model and literature values of the rate constants supports the credibility of the key steps in the proposed mechanism.

Figure 7 shows the fit for the formation of BMPO–OH is within the standard deviation of the experimental data for both the BQ-only solution and 1:10 BQ/LVG mixture. The fit for the BMPO–H formation is also reasonable for both solutions, especially at later times when the concentration increases. The model fit is worse for BMPO–H formation during the first 1500 s when the concentration of BMPO–H is low relative to the limit of detection (LOD) of the EPR. The ability of the model to reproduce both BMPO–OH and BMPO–H formation simultaneously with reasonable accuracy indicates the proposed combination of the BQ irradiation mechanism and computational study mechanism for H^•^ generation is consistent with the experimental results.^35,36,47^

The kinetic model was also used to investigate the formation of HO_2_^•^ in the mixture as it is expected to form through R11. The BMPO–OOH adduct was detected in the mass spectra (Figure S5D) but was not observed in the EPR spectra. In the presence of BMPO, the model predicts that BMPO–OOH reaches a maximum concentration of ∼0.9 and 1.0 μM for the pure BQ solution and 1:10 BQ/LVG mixture, respectively (Figure 7C,D). While this concentration is above the LOD for the EPR experiment (∼0.25 μM), it is still relatively low in comparison to the other radical species present. Additionally, the BMPO–OOH signal overlaps with peaks already present for the other radical species, especially BMPO–OH, which has a maximum concentration 100 times greater than the predicted concentration of BMPO–OOH, so the contribution of BMPO–OOH to the overall EPR spectra may be swamped by that from other radicals. The model also predicts the loss of dissolved oxygen during the irradiation would be less than ∼8% of its original concentration, resulting in a final O_2_ concentration to be 50 times higher than the concentration of BMPO–H detected. This suggests that the depletion of dissolved oxygen would not limit the formation and detection of HO_2_^•^.

We conducted a sensitivity analysis of the model by varying rate constants. The model found that reactions R1–R7, R9, R11, R23, R25–R28, and R30 are all crucial for accurately modeling the BMPO–OH, BMPO–H, and BMPO–OOH formation over time. This primarily agrees with literature mechanisms describing BMPO–OH formation.^35,36^ It also suggests that it is important to include the proposed photolysis of BQH^•^ to form H^•^ (R7) in order to predict the formation of BMPO–H (R26) detected by EPR and the subsequent formation of BMPO–OOH (R11 and R28) observed in the mass spectra. Based on the outputs of the kinetic model, R8, R10, R12, R24, and R29 and all the ROS coupling reactions (R13–R22) contribute negligibly to the concentration of BMPO–OH, BMPO–H, and BMPO–OOH. The insignificance of R8 and R24 suggests that the only meaningful reaction pathway for BQOH^•^ is R6 to form BQH^•^, the necessary precursor for H^•^ formation (R7). The insignificance of R10 suggests that the only relevant sink for LVG is through the reaction with ^•^OH (R9), although the rate constant for R10 (7.53× 10^8^ M^–1^ s^–1^) is within the same range as the rate constants of organic molecules with triplet-excited states previously compiled.^9,64^ The little sensitivity of R12 indicates that the primary reaction pathway for H^•^ is through R11 to form HO_2_^•^ in the absence of BMPO. The insignificance of the ROS coupling reactions suggests all the ROS formed in the solution are more likely to react with the other species than each other. Specifically, the ^•^OH radical readily reacts with BQ (R5) and LVG (R9) to form organic radicals and with BMPO (R23) to form BMPO–OH.

**Figure 8:**
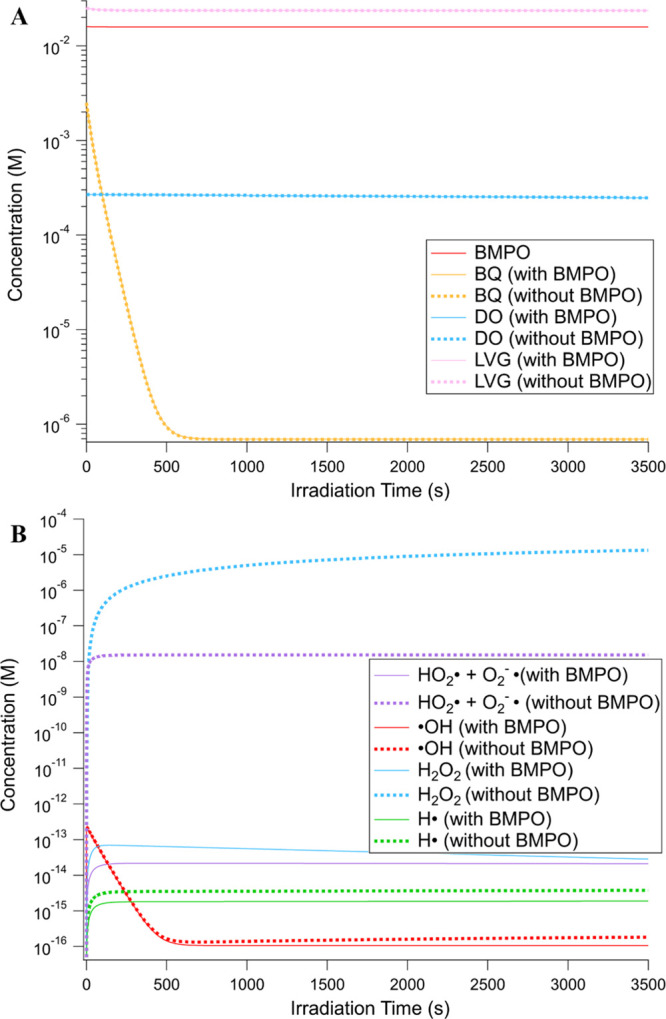
Model-predicted time evolution of concentrations of (A) reactants (BQ, LVG, DO, and spin trap BMPO) and (B) ROS (^•^OH, H_2_O_2_, H^•^, and O_2_^•–^/HO_2_^•^) in the 1:10 BQ/LVG mixture with and without BMPO. Note that HO_2_^•^ and O_2_^–•^ are plotted together as they form the same BMPO–OOH adduct.

Figure 8A shows that there is a substantial decrease in the concentration of BQ over time, which correlates with the formation of hydroxylated BQ derivatives (Figure S6). This supports our assumption from the radical yield calculation (Figures 3 and 4) that the concentration of BQ and its derivatives remains constant overall, but the calculation also assumes that these derivatives have similar absorption properties to BQ, which could not be verified experimentally. The rapid conversion of BQ to its derivatives suggests that the radical yield calculation (Figures 3 and 4) is only likely to hold at early irradiation times, but it is still useful for qualitatively comparing the radical formation between samples. In contrast, the concentrations of BMPO and dissolved oxygen change very little over time. This verifies that BMPO is present in sufficient excess that its depletion over time does not impact adduct formation in the system. The model predictions also align with the dissolved oxygen measurements that found that the irradiation of the mixture leads to a negligible decrease in dissolved oxygen, despite the formation of HO_2_^•^ occurring in the solution. This indicates that there is sufficient dissolved oxygen to react with H^•^ to form HO_2_^•^. Figure 8A also shows minimal depletion in LVG concentration in the 1:10 mixture (approximately 6%) over the course of irradiation. However, in the 1:1 ratio of LVG, the model predicts that the concentration of LVG decreases by about 17% (Figure S7). This agrees with previous studies, suggesting that the reaction with the aqueous ^•^OH (R9) is a noteworthy sink of LVG and can impact the overall concentration of LVG when it is not present in excess of ^•^OH.^38−40^ This further supports that LVG loss through aqueous ^•^OH oxidation needs to be considered when using it as a tracer molecule for BBOA measurements.

The kinetic model was also used to predict the time evolution of species that are not detectable via EPR in the 1:10 BQ/LVG solution both with and without BMPO (Figures 8 and S6). Figure 8B shows the concentration of ^•^OH ranges from 10^–13^ to 10^–16^ M, which are in a very similar range to previously reported concentration ranges for cloud droplets and particulate matter.^65^ Additionally, it shows that, in the absence of BMPO, there is no significant change in the concentration of ^•^OH, suggesting that the reactions with BQ and LVG are more important sinks for ^•^OH than the formation of BMPO–OH. This is because the rate constant of ^•^OH with BMPO (R23) is about 50 times lower than the rate constant for the reaction of ^•^OH with BQ (R5) and 10 times slower than its reaction with LVG (R9). This means that the measured concentration of trapped ^•^OH shown in Figure S3 would represent the lower limit of the formed ^•^OH in the solution. In contrast, in the absence of BMPO, the total superoxide concentration would increase by 6 orders of magnitude to over 10 nM, suggesting efficient trapping by BMPO and more generation of HO_2_^•^ via the reaction of dissolved oxygen and H^•^. Additionally, without BMPO, the concentration of H_2_O_2_ would increase by 8 orders of magnitude to 10 μM because the reaction between HO_2_^•^ and O_2_^–•^ (R22) becomes the dominant sink for the superoxide radical in the solution to produce H_2_O_2_.

## Conclusions

This study examined the effect of irradiation on mixtures containing BQ and LVG and BBOA tracer molecules, using EPR, HRMS, and kinetic modeling to gain insight into radical formation and changes in chemical composition over the course of photochemical aging. The EPR analysis showed that the irradiation of these mixtures leads to the formation of ^•^OH, H^•^, and organic radicals, which can perpetuate radical chain mechanisms within aqueous droplets. The formation of ^•^OH from the irradiation of aqueous BQ has been previously observed,^35,36,52^ and this study supports that BQ can be a significant aqueous-phase source of ^•^OH. The prevalence of this mechanism under different atmospheric conditions including different light intensities, and more complex mixtures should be explored in future work to gain better insight into the broader impacts of this mechanism on aerosol composition. Additionally, it would be worthwhile to incorporate this mechanism into the aerosol process and large-scale models, such as CMAQ, to evaluate its contribution to the role in the overall atmospheric ^•^OH budget. A previous computational study proposed that H^•^ comes from the photochemical decomposition of semiquinone radicals,^47^ but to our knowledge, this is the first experimental observation of H^•^ formation from aqueous BQ. The detection of H^•^ suggests the potential formation of HO_2_^•^ through reactions with dissolved oxygen, which was confirmed by HRMS. The mass spectrometric analysis provided a way to distinguish BMPO adducts containing BQ and LVG derivatives, confirming that aqueous photochemistry can transform innocuous chemicals, like LVG, into organic radicals that lead to increasingly complex chemistry within the droplets. Additionally, the mass spectra of the irradiated BQ + LVG mixtures contained a large number of hydroxylated benzoquinone derivatives, highly oxidized organic molecules, and oligomers that can enhance SOA formation.^9,23,31,32^ This implies that quinones such as BQ may substantially impact photochemically driven reactions in biomass-burning plumes, although future experiments should explore the products of aqueous photochemistry of quinones in the presence of other relevant BBOA molecules such as humic-like substances, phenolic compounds, and nitrogen-containing compounds.^66,67^ The results of the kinetic model show that ^•^OH rapidly reacts with LVG and can shorten its atmospheric lifetime, which has important implications for its application as a tracer molecule in field studies. The kinetic model also predicts that systems without BMPO will contain elevated concentrations of HO_2_^•^ and H_2_O_2_. As inhalation of ROS can cause oxidative stress in the lungs,^68,69^ our results imply that irradiation may impact the toxicity of BBOA particles,^68^ although this aspect would need to be further investigated by dedicated studies including cell exposure and toxicity assessments.

## Supporting Information Available

Reactions 25 and 30’s rate constant determination; dissolved oxygen measurements; EPR fitting parameters; average and standard deviation for MCGA-determined rate constants; mercury lamp spectral flux; EPR spectra for 1:1 BQ/LVG and 1:100 mixtures; non-normalized radical formation over time; BMPO mass spectrometry ionization mechanisms; mass spectra; and modeled concentrations over time for BQ derivatives (PDF)
