# Supplementary material for: Photoenhanced Radical Formation in Aqueous Mixtures of Levoglucosan and Benzoquinone: Implications to Photochemical Aging of Biomass-Burning Organic Aerosols
Source: J Phys Chem A. 2023 Jun 7;127(24):5209–21. doi: 10.1021/acs.jpca.3c01794 (PMC10291547; doi:10.1021/acs.jpca.3c01794)
Supplement: Supplementary file 1 — jp3c01794_si_001.pdf [file jp3c01794_si_001.pdf]

Supporting Information for

Photoenhanced Radical Formation in Aqueous Mixtures of Levoglucosan and Benzoquinone: Implications to Photochemical Aging of Biomass Burning Organic Aerosols

Lena Gerritz, Meredith Schervish, Pascale S. J. Lakey, Tim Oeij, Jinlai Wei, Sergey A. Nizkorodov, Manabu Shiraiwa

Department of Chemistry, University of California Irvine

Contents

Rate Constant of R25 and R30 2
Dissolved Oxygen Measurements 2
Table S1 3
Table S2 4
Figure S1 5
Figure S2 6
Figure S3 7
Figure S4 8
Figure S5 9
Figure S6 10
Figure S7 11

Rate Constant of R25 and R30

The rate constants for the decomposition of BMPO—OH (R25) and BMPO—OOH (R30) were previously measured under dark conditions to be around 6×10^{-4} and 7×10^{-4} s^{-1}, respectively.[1,2] There are no literature values for these rate constants under UV irradiation and at pH~5. The rate constant for reaction 25 was calculated using MCGA to fit the experimental EPR data using a range of 5 - 100 × 10^{-4} s^{-1}.

The rate constant for R30 was estimated based on the detection limitations of the EPR and HRMS analyses. BMPO—OOH was not detected using EPR so there were no data available to estimate the rate constant using MCGA. HO_2• is expected to form through R11, and BMPO—OOH is detected using mass spectrometry, so it is necessary to include it in the kinetic model. The concentration of BMPO—OOH may be above the limit of detection for EPR, but its signal overlaps significantly with the much stronger signal from BMPO—OH. This prompted us to estimate the concentration of BMPO—OOH required to visibly distort the signal of BMPO—OH as an upper bound for the model, rather than using the limit of detection of the EPR. Through signal simulations for the 1:10 BQ:LVG mixture, we found that the BMPO—OH peaks are detectably distorted when the concentration of competing signals reaches around 35% of the concentration of BMPO—OH, around 1.4×10^{-6} M. This was used as the estimated maximum concentration of BMPO—OOH that could be present in the system before its effects would be noticeable. The minimum concentration of BMPO—OOH was estimated based on the relative intensity of BMPO—OOH fragment relative to BMPO—OH fragment detected by mass spectrometry, around 8% (0.32×10^{-6} M). This cannot be used as an actual estimate of the BMPO—OOH concentration because of differences in the ionization efficiency for each species, but it provides a reasonable lower bound estimate for the purposes of this kinetic model. Using a maximum BMPO—OOH concentration range of 0.3-1.4× 10^{-6} M, the bounds of R30 were determined to be 2.2-9.5× 10^{-3} s^{-1} yielding concentrations of 1.4× 10^{-6} M and 0.32×10^{-6} M, respectively.

Dissolved Oxygen Measurements

A MI-730 Micro-Oxygen electrode (Microelectrodes, Inc) with an O2-ADPT Oxygen Adapter (Microelectrodes, Inc.) was used to determine the concentration of dissolved oxygen in mixtures before and after irradiation. The electrode was calibrated before each experiment using Milli-Q water purged with clean air (21% O_2) creating a solution in a solubility equilibrium with

air and purged with nitrogen to create an oxygen-free solution. Based on the Henry's law constant of 1.3×10^{-3} mol kg^{-1} atm^{-1}, the concentration of dissolved oxygen in the aerated solution was assumed to be 0.27 mM. To account for the probe's significant temperature sensitivity, all solutions were placed in a large water bath at 20°C for at least 10 min prior to the dissolved oxygen measurements. All measurements were performed in triplicate to account for probe sensitivity.

Prior to irradiation, samples of 5 mM BQ were purged with clean air in a cuvette for approximately 15 min to ensure that the dissolved oxygen concentration reached a solubility equilibrium. The initial dissolved oxygen reading was recorded. The cuvettes were capped and sealed with parafilm to ensure the system is closed. The samples were then irradiated for between 0-30 min using a Xenon Arc lamp and placed in the room temperature water bath until they reached room temperature. The dissolved oxygen level of the sample after irradiation was then measured again. The change in dissolved oxygen concentration was measured to be less than 10% after half an hour of irradiation.

Table S1: EPR fitting parameters used for each BMPO-radical adduct.

**Radical**	**g-factor**	**Hyperfine splitting constants (α,β,γ)**
BMPO—OH (1)	2.0063	14.18, 15.33, 0.58
BMPO—OH (2)	2.0063	14.14, 12.61, 0.69
BMPO—R	2.0063	14.75, 21.19
BMPO—OR	2.0063	14.76, 17.28
BMPO—H (1)	2.0061	15.59, 20.82
BMPO—H (2)	2.0061	15.59, 22.29

Table S2: Average rate constants and standard deviations taken over 24 data sets determined by MCGA.

**Rxn #**	**k (average) ± standard deviation**
R1	1.69 (± 0.10) × 10^{-2} s^{-1}
R2	2.84 (± 0.90) × 10^{12} s^{-1}
R3	9.79 (±7.44) × 10^{6} s^{-1}
R4	1.77 (± 0.90) × 10^{9} s^{-1}
R5	2.42 (± 0.99) × 10^{10} $M^{-1}s^{-1}$
R6	1.04 (± 0.45) × 10^{10} $M^{-1}s^{-1}$
R7	5.30 (± 1.63) × 10^{-6} s^{-1}
R8	1.27 (±0.89) × 10^{8} $M^{-1}s^{-1}$
R9	3.60 (±8.32) × 10^{8} $M^{-1}s^{-1}$
R10	7.70 (±4.67) × 10^{8} $M^{-1}s^{-1}$
R11	Fixed
R12	Fixed
R13	Fixed
R14	Fixed
R15	Fixed
R16	Fixed
R17	Fixed
R18	Fixed
R19	Fixed
R20	Fixed
R21	Fixed
R22	Fixed
R23	4.52 (± 1.83) × 10^{8} $M^{-1}s^{-1}$
R24	3.72 (± 3.21) × 10^{6} $M^{-1}s^{-1}$
R25	1.74 (± 0.17) × 10^{-3} s^{-1}
R26	2.01 (± 0.86) × 10^{8} $M^{-1}s^{-1}$
R27	4.94 (±1.37) × 10^{-4} $M^{-1}s^{-1}$
R28	Fixed
R29	Fixed
R30	Fixed

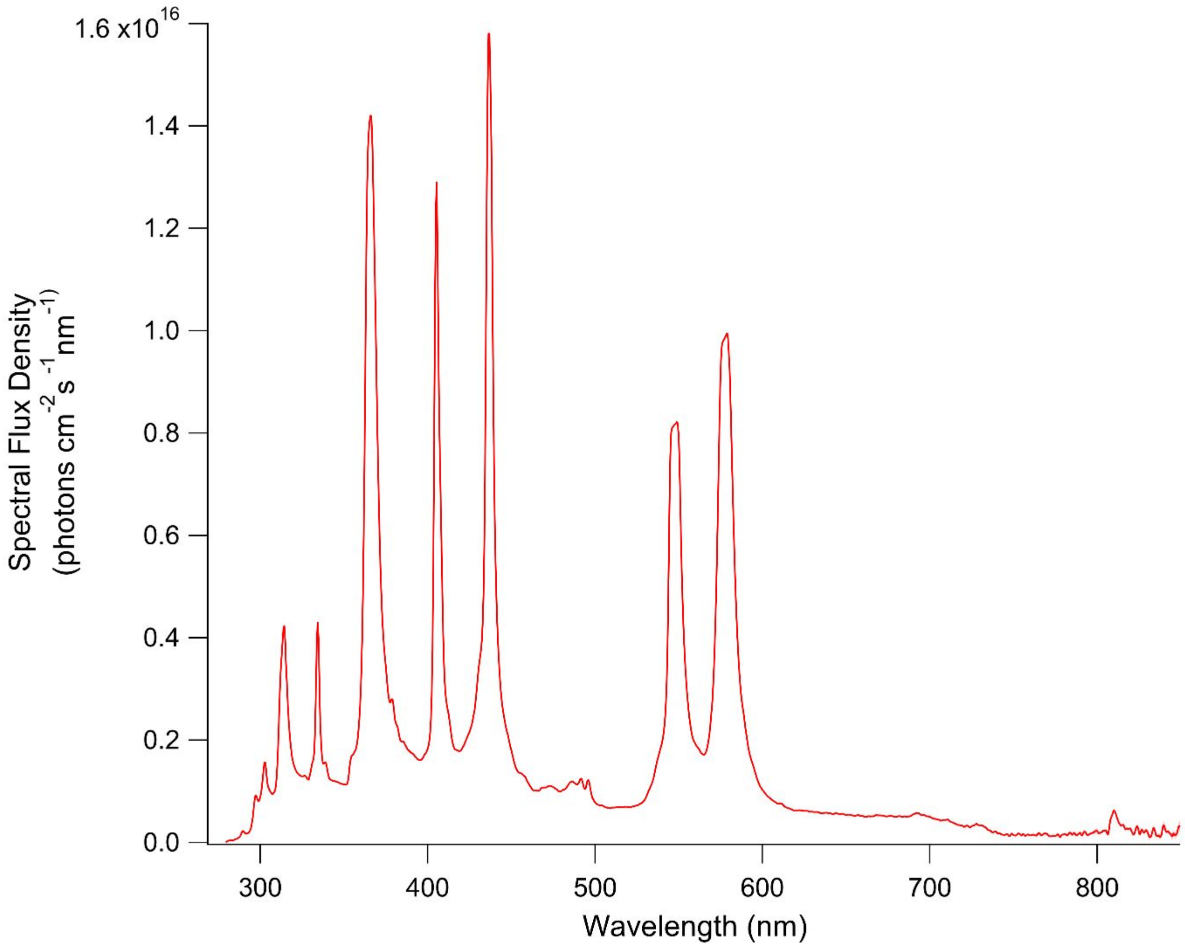

Figure S1: Spectral flux density of mercury lump measured at the location of the irradiated solution with a calibrated spectroradiometer.

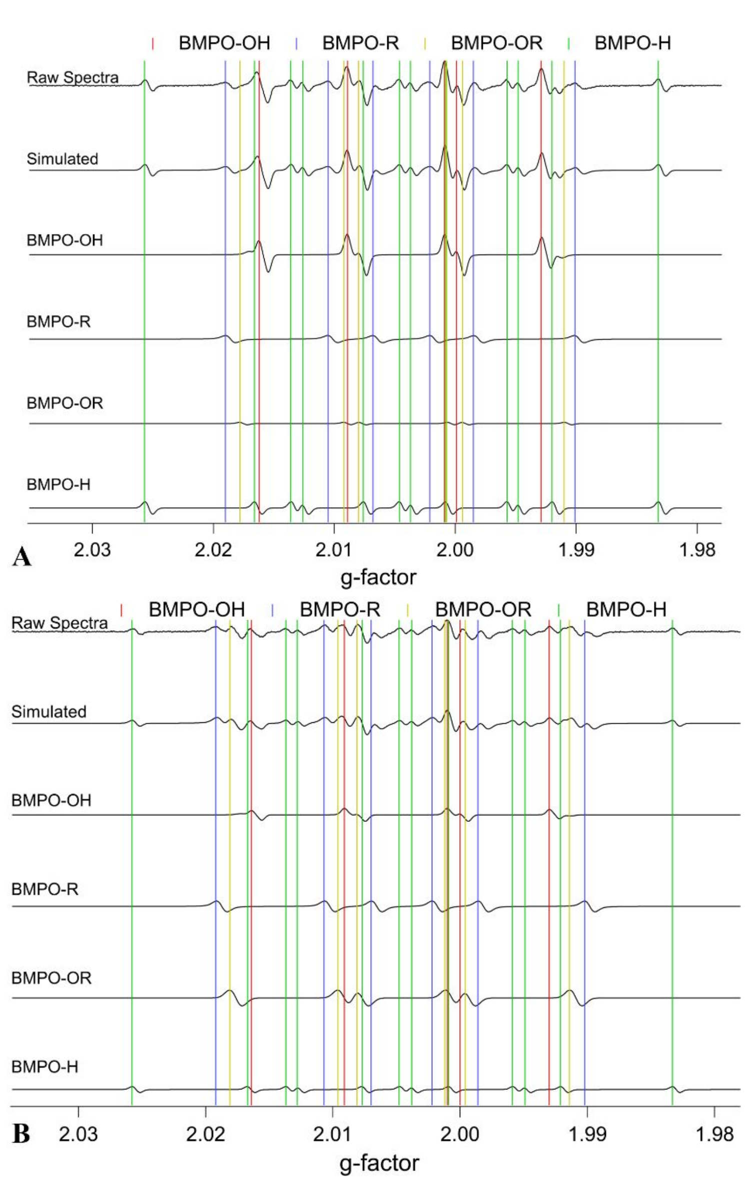

Figure S2: EPR spectra for surrogate mixtures after 53 min of irradiation for A). 1:1 BQ:LVG B). 1:100 BQ:LVG. The observed spectra were deconvoluted based on radical species trapped by BMPO (BMPO—OH: red, BMPO—R: Blue, BMPO—OR: Yellow, BMPO—H: Green).

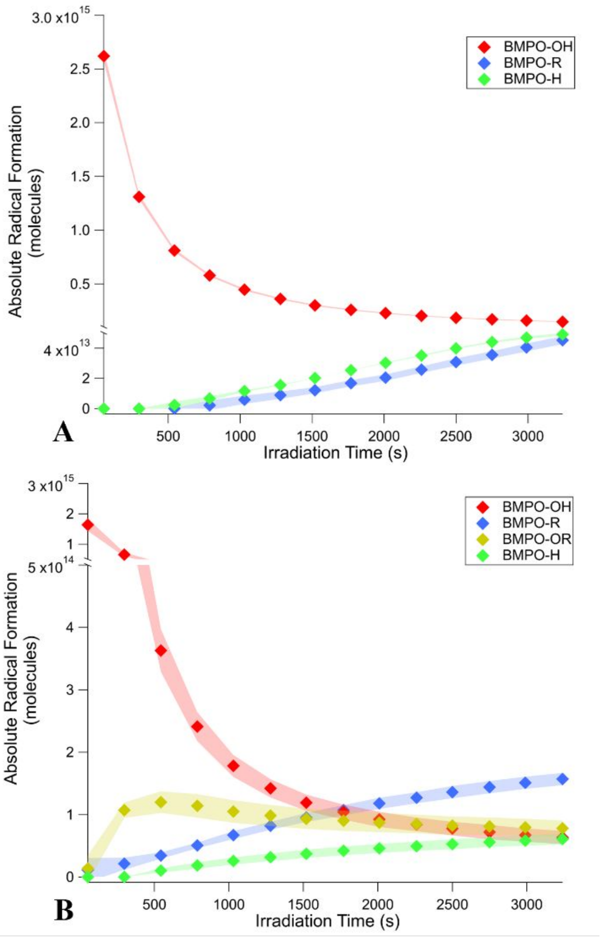

Figure S3: Absolute number of BMPO-radical adducts formed over irradiation time for solutions containing 2.5 mM of BQ solutions A) without LVG and B) 1:10 BQ:LVG mixture with >10 mM BMPO. Note the split axes.

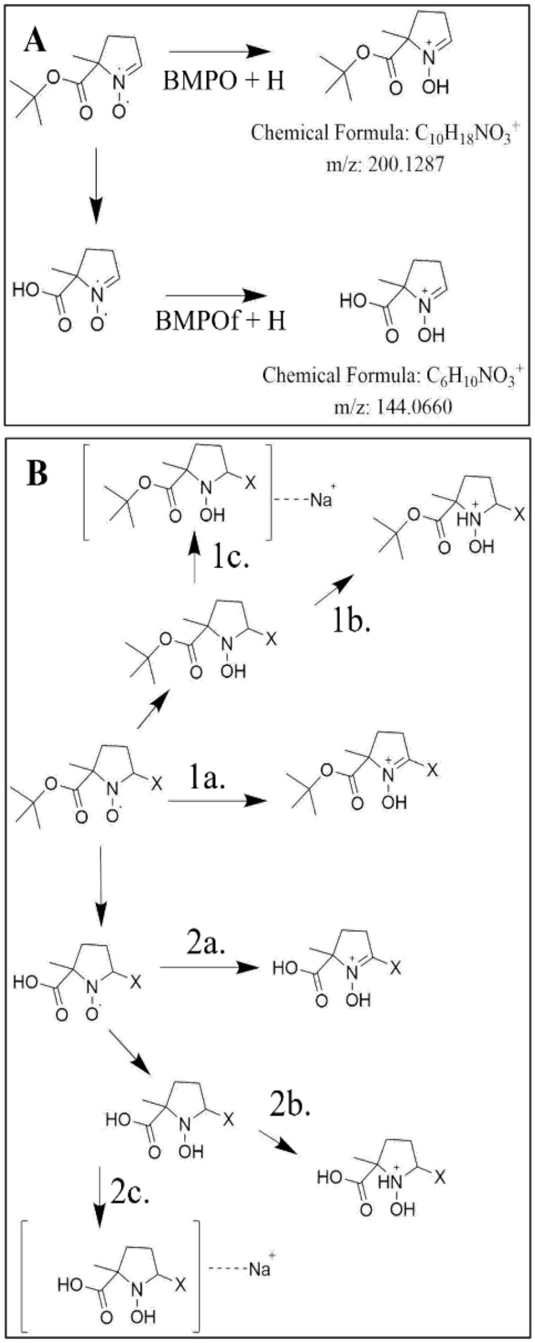

Figure S4: A). BMPO and BMPO fragment (BMPOf) positive ion mode mass spectrometry ionization pathways and B). BMPO—X positive ion mode mass spectrometry ionization pathways where X is any radical adduct.

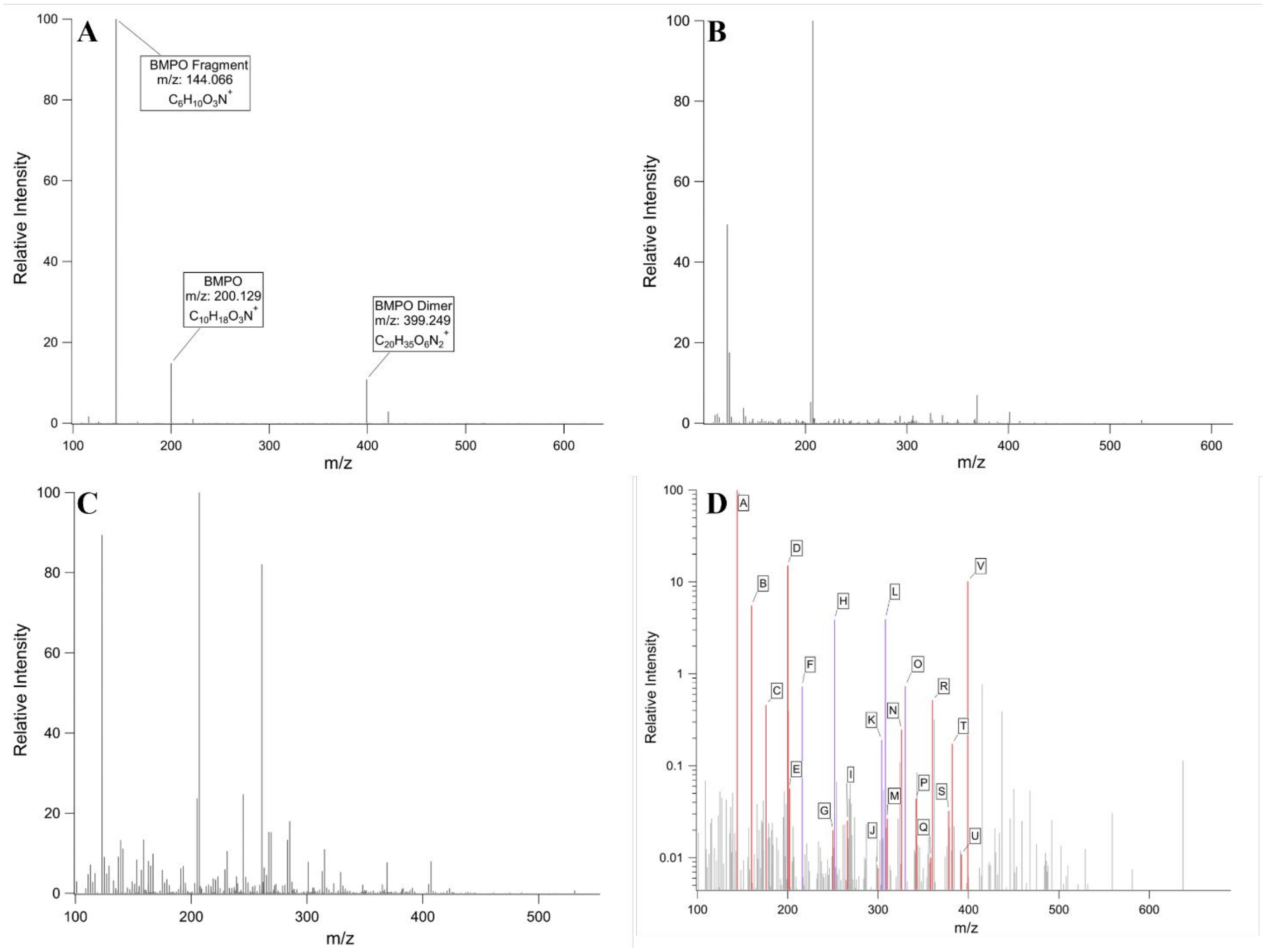

Figure S5: Positive ion mode ESI mass spectra for the 1:10 BQ+LVG mixture A.) Non-irradiated mixture with BMPO integrated over the retetion time of BMPO. B.) Non-irradiated mixture with BMPO integrated over the entire chromatogram C.) Mixture irradiated for 10 min without BMPO D.) Mixture with BMPO irradiated for 10 min labeled with key BMPO adduct peaks (red) and other major peaks (purple). Assignments above *m/z* 400 were not attempted due to the complexity of the ionization and fragmentation mechanisms.

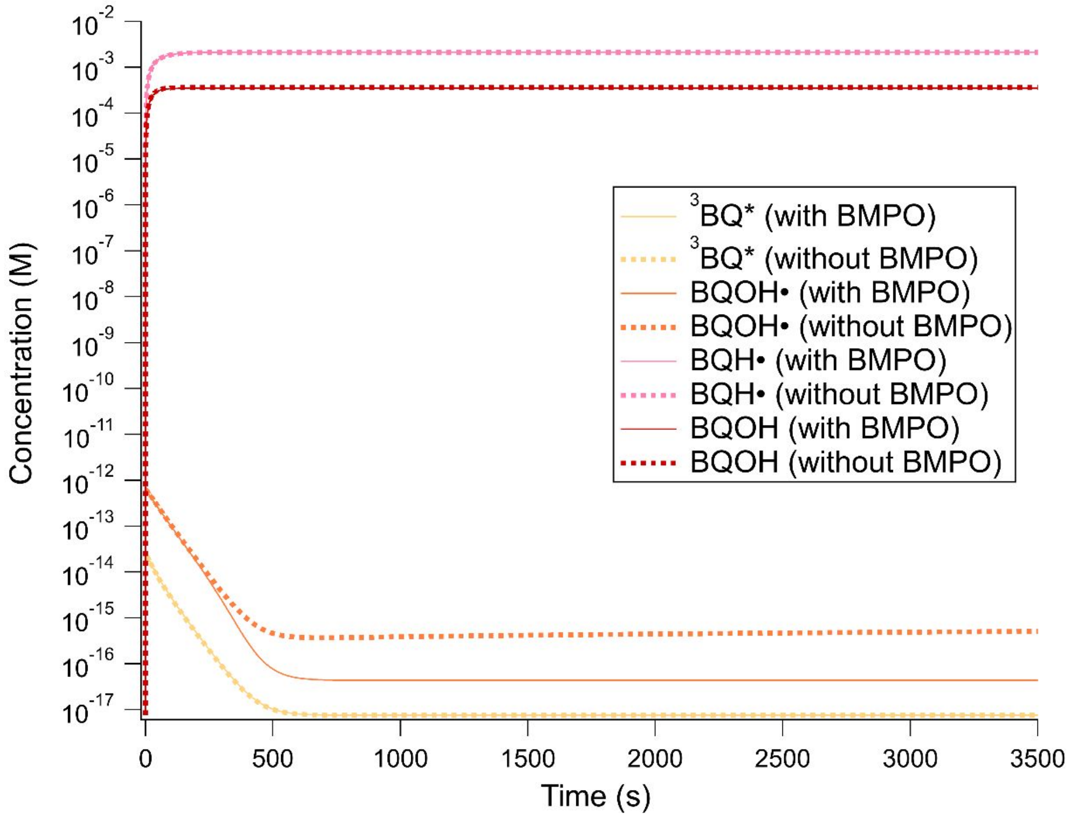

Figure S6: Model predicted concentrations of 3BQ*, BQOH•, BQH•, and BQOH over irradiation time in the 1:10 BQ:LVG surrogate mixture with (solid lines) and without (dashed lines) BMPO.

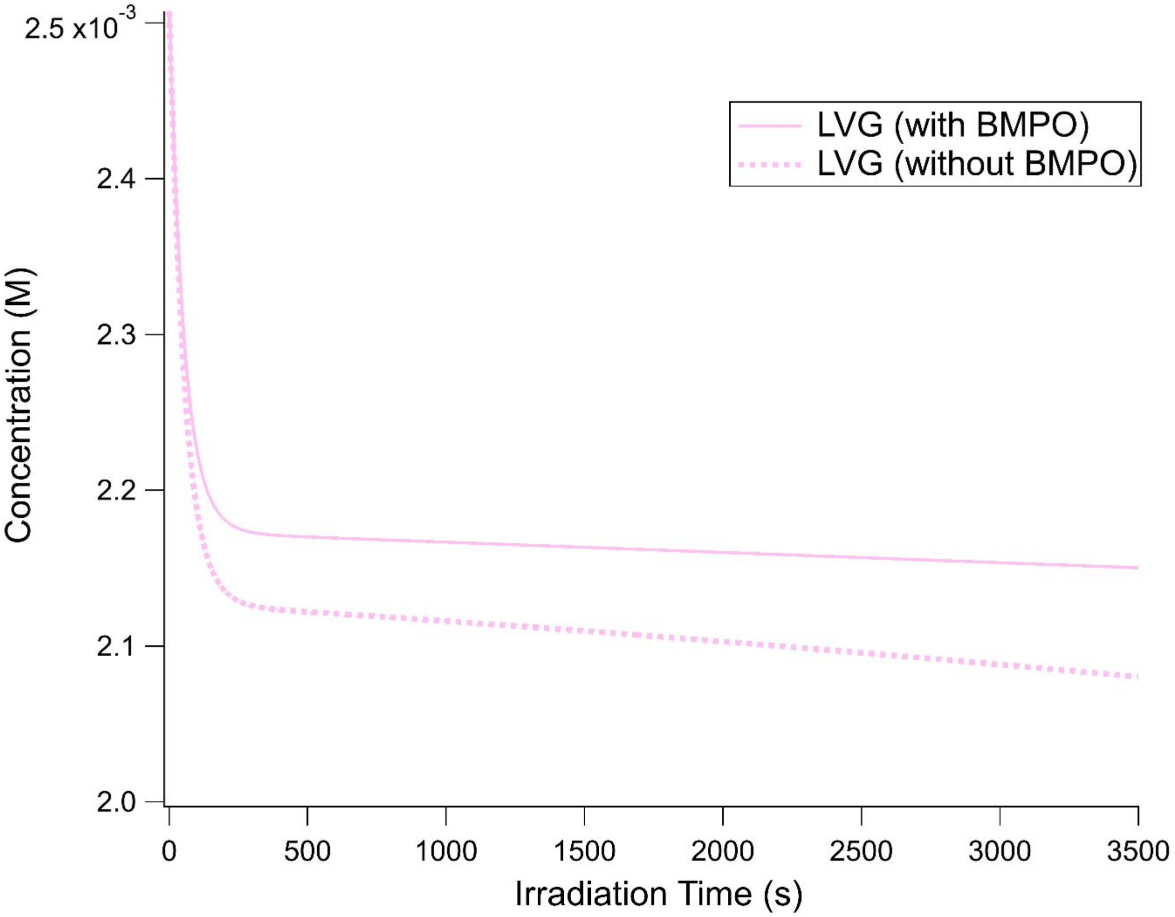

Figure S7: Model-predicted time evolution of the LVG concentration over time in mixture containing 1:1 BQ:LVG with and without BMPO.
